# Heat Stress Response to National-Committed Emission Reductions under the Paris Agreement

**DOI:** 10.3390/ijerph16122202

**Published:** 2019-06-21

**Authors:** Fang Wang, Jintao Zhang

**Affiliations:** 1 Department of Climate and Environment Change, Institute of Geographic Sciences and Natural Resources Research, Chinese Academy of Sciences, Xicheng District, Beijing 100864, China; zhangjt.17s@igsnrr.ac.cn; 2 Key Laboratory of Land Surface Pattern and Simulation, Chinese Academy of Sciences, Xicheng District, Beijing 100864, China; 3 College of Resources and Environment, University of Chinese Academy of Sciences, Xicheng District, Beijing 100864, China

**Keywords:** heat stress, INDCs, Paris Agreement, exposure

## Abstract

With the changes in global temperature and humidity, heat stress is expected to intensify in the coming decades. Under the scenario that greenhouse gas emissions keep increasing until the end of this century, there is the possibility of extensive global exposure to high heat stress. While under new mitigation efforts (as part of the Paris Agreement, signatory nations pledged to implement the Intended Nationally Determined Contributions (INDCs) for emission reductions), the regional response of heat stress to pledged emission reductions remains unclear. In this study, we analyze the heat stress response in global hotspot regions, targeting emission scenarios resulting from the INDCs pledges. Our study revealed that under the INDCs-continuous mitigation, the heat stress effect in global hotspot regions (North China, South Asia, and the Amazon) is estimated to be lower than 29 °C in the next three decades and to be from >33 °C to less than 30 °C to this century end. The heat stress effect indicates a great reduction at the continuous mitigation compared with the delayed mitigation, and the population exposed to dangerous heat stress would also decrease approximately one order of magnitude. If limiting warming to a lesser amount (1.5/2 °C targets), significantly further reduction of the population exposed to heat stress in the middle and low latitudes can be achieved, thus avoiding the adverse effects associated with heat stress. Therefore, the national intended mitigation actions under the Paris Agreement will play a crucial role in reducing the heat stress risk in these hot and humid regions. These findings will help to improve the understanding of the future risks of heat stress and are crucial for mitigation and adaptation actions in hotspot areas (approximately 1/3 of the world’s population).

## 1. Introduction

Climate change can increase the risk of exceeding the human thermoregulatory capacity [1,2,3,4,5,6,7]. When the ambient temperature increases, the human body feels increasingly uncomfortable. Some studies showed that under the RCP 8.5 (Representative Concentration Pathway) emission scenario, many regions, including China, Eurasia, Africa, and Latin America, are vulnerable to heat stress [8]; furthermore, due to heat stress, the labor force during the summer’s peak period could be reduced by more than 60% [8]. To date, there have been a number of deaths due to extreme heat waves, such as the 2003 European heat wave that killed tens of thousands of people [9].

Human health is affected by temperature and humidity. The human body can effectively dissipate heat through evaporative cooling even under high-temperature conditions, if the moisture level is low. However, in hot and humid conditions, the efficiency of evaporative cooling slows, and the body may not be able to maintain one stable core temperature. The body may accumulate heat and suffer from fever [10,11,12]. The increase in near-surface air temperature causes a change in humidity; the combined changes in both the temperature and humidity pose a major threat to human health [8,13,14]. The heat stress index, the wet bulb temperature (also abbreviated as Tw), can be used to measure the potential impact of heat on the human body. It captures the comprehensive effects of air temperature and humidity on the heat exchange between the environment and human body. Over the past four decades, the wet bulb temperature has been shown to increase with temperature and humidity changes [12,15,16].

Increasingly more studies use heat stress as a human health risk indicator. The frequency of these events may increase the most in low-latitude and mid-latitude regions in the future [17]. In this paper, the 14 generalized circulation models (GCMs) from the fifth phase of the Coupled Model Intercomparison Project (CMIP5) were used to perform the global analysis of population exposure to extreme wet-bulb temperature under the multi-emission scenarios presented in the Paris Agreement (the INDC (Intended Nationally Determined Contribution) emission reduction scenario and global temperature control scenarios, 1.5 °C and 2 °C). This analysis also integrates with population predictions from SSPs (shared socioeconomic pathways). We calculated the changes in daily air temperature, humidity, and wet-bulb temperature caused by various emission scenarios of the Paris Agreement and evaluated the risk differences in heat stress between the INDC emission reduction scenario and the global temperature control scenarios (1.5 °C and 2 °C).

## 2. Data and Methods 

### 2.1. Emission Scenarios

The emission scenarios used include two categories: One is the temperature control scenario under the Paris Agreement, i.e., the global target of a 1.5 °C scenario and the global target of a 2 °C scenario; the other is the INDC pledged scenario. 

The global targets of the 2 °C and 1.5 °C scenarios are based on the assumption that global mean warming to this century end would be limited to well below 2 °C or 1.5 °C. These are idealized global scenarios and they are presented in the IPCC (Intergovernmental Panel on Climate Change) Assessment Report 5 and the 1.5 °C Assessment Report [18,19], respectively. The 1.5 °C or 2.0 °C warming condition is defined by projections in which the 9-year smoothed temperature reaches 1.5 °C or 2.0 °C warming relative to the preindustrial level in each climate model. A total of 46 simulations is used for 1.5 °C warming and 42 simulations for 2 °C warming. The multi-model ensemble mean is calculated for 1.5 °C and 2 °C.

**Figure 1:**
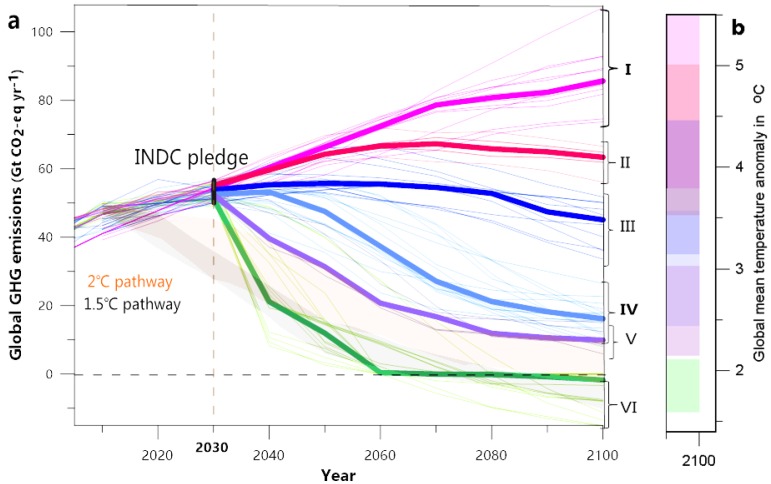
Global greenhouse gas emissions for INDC (Intended Nationally Determined Contributions) scenarios. (**a**) Future emission pathways analyzed in this study. Emission scenarios consistent with INDC pledges by 2030 are collected from the UNFCCC (United Nations Framework Convention on Climate Change) database. The INDC emission targets are calculated based on each country’s data and are estimated to be approximately 50–56 Gt CO2 for 2030. After 2030, various policy assumptions are grouped into six categories and different color lines display the extended emissions for the post-2030 period. Thick lines indicate the median emissions level for each group (I, II, III, IV, V, and VI). Group I (baseline scenario) contains scenarios without any additional climate policies or mitigation actions, where the greenhouse gas emissions continue to increase according to current trends. Group II is similar to the baseline scenario but allows for lower energy intensity in the future. Group III considers existing climate policies, a weak interpretation (e.g., 2020 Copenhagen Pledges), and extrapolation of these targets beyond 2020 based on emissions intensity. Global emissions were assumed to peak in 2030 in Groups IV to VI. Specifically, Group IV may be described as a “continued action” pathway. The relatively constant decarbonization rates were approximately followed for the period after 2030. The overall trend of Group V is similar to Group IV, but more rapid mitigation after 2030 is the distinguishing characteristic. Group VI involves CCS (Carbon Capture and Storage) action accelerating decarbonization and determining negative emissions in some pathways. The range of the 1.5 °C and 2 °C pathways are plotted for reference, in grey and orange shaded areas, respectively. (**b**) The estimates of global mean warming above the pre-industrial level (ΔT) for each scenario group (uncertainty range of 33–66% in color shaded areas).

The INDCs pledges were submitted by 192 countries. The national INDC pledge has been continuously updated. This study analyzed 165 national INDC pledges submitted as of May 2019, in which the European Union (EU) member states submitted an INDC pledge for the entire region. The INDC pledge of each country may be found on the United Nations Framework Convention on Climate Change (UNFCCC) website [20,21]. The emission targets of countries range from absolute values to relative values of the baseline year level or the emission reduction targets relative to the baseline year level. We analyzed and extracted each country’s emission targets. The annual global emissions pathways were obtained using a linear interpolation between the last historical year and committed years (the 2030 year). For the post-2030 period, annual emission pathways were obtained by extending the continued action of emission reduction under INDC scenarios (Figure 1a). For additional information on the INDC dataset, please refer to Supplementary Material Table S1 and the study of Wang [22,23] (Table S1). In our discussion of the heat stress response in the global INDC scenario, we used the INDC ‘continuous-action’ scenario and the INDC ‘delayed-action’ scenario. The first group (I) in Figure 1 is referred as the “delayed action” scenarios, and the scenarios are emission projections that assume that the INDC mitigation action delayed or stalled after 2030 and return to a no-policy trajectory. The third and fourth groups (III, IV) are referred as the extended “continuous action” (INDC commitment) scenario, and the scenarios are based on the assumption that the national INDC actions of emission reduction will continue to the late 21st century and global emissions will decline steadily. The relatively constant decarbonization rates were followed for the period after 2030.

### 2.2. Estimation of Heat Stress Response to Emissions

#### 2.2.1. Heat Stress Index Calculation

Heat stress is calculated based on the wet-bulb temperature ($T_{w}$). The daily maximum $T_{w}$, $T_{wmax}$, is calculated according to the algorithm described by Tsonis [24] and Davies-Jones [25]. The calculation of $T_{wmax}$ is based on the following formula:

(1) $$e=e_{s}\left(T_{w}\right)-\frac{c_{pd}p}{\varepsilon L_{v}}\left(T-T_{w}\right)=e_{s}\left(T_{w}\right)-Ap\left(T-T_{w}\right)$$

where *T* is dry bulb temperature; *T_w_* is wet-bulb temperature; *c_Pd_* is isobaric heat capacity of dry air; *A* and ε are constants; *p* is pressure; *L_v_* is latent heat of vaporization of water; and *e_s_* is saturation water pressure. The integral expression of *e_s_*(*T*) is as follows:

(2) $$e_{s}\left(T\right)=e_{so}\exp\left[\frac{L_{v}}{R_{v}}\left(\frac{1}{T_{0}}-\frac{1}{T}\right)\right]$$

where *e_so_* is saturated vapor pressure at *T*_0_ (273.15 k). All these data are from the simulation results obtained for 14 CMIP5 GCMs (Supplementary Table S1).

#### 2.2.2. Estimated Heat-Stress Change under the Paris Agreement

Heat-stress change under the Paris Agreement is estimated based on the following:

- Firstly, to assess the global mean warming level induced by each greenhouse gas emission scenario. Based on the 78 climate sensitivity experiments from the earth system models (ESMs) ensemble of CMIP5 [26], we assessed the possible corresponding global mean temperature rise for various emission scenarios [22,23]; we also integrated several other studies (temperature rise levels for some pathways have been provided) [27,28,29]. After a comprehensive assessment, we determined the most likely range of temperature increase for the continuous and delayed mitigation pathways of INDCs (Figure 1b).
- Secondly, to estimate the space pattern of heat-stress change. We used 14 ESMs from the CMIP5 archives. These models have different levels of climate sensitivity [30] and represent a wide range of climate responses to emission scenarios. Supplementary Table S2 summarizes information regarding the 14 ESMs used in this study. All model data were interpolated to a common 1.5° × 1.5° horizontal grid. The spatial pattern of the heat stress in response to each scenario was identified using a time-slice approach, where the spatial state at a specific warming point related to $∆T_{INDC}$ (and the global targets of the 1.5 °C and 2 °C scenarios) was separately obtained from the decadal time slices with the respective mean warming for each model. The spatial simulations of future heat-stress change were based on the CMIP5 models ensemble and the 5–95th percentile confidence intervals (CIs) were estimated from 10,000 bootstrapped subsamples of data. The period of 1980–2010 was referred to as the present-day baseline and the preindustrial period was defined as 1861–1900.
- Lastly, to evaluate population exposure to extreme heat stress. We used the population data from socioeconomic development scenarios of SSPs [31] and the data were changed to a 1.5 ° **×** 1.5° latitude/longitude grid to match the GCM resolution. The population exposed to an extreme wet-bulb temperature was calculated at a daily time resolution. If the wet-bulb temperature at a given grid cell exceeded the threshold value on a given day (for example, a wet-bulb temperature of 32 °C or 30 °C), then the grid cell was considered to be exposed, and the total number of exposed cells was counted in units of person-days. The total amount of annual exposure (in units of person-days) is the number of people multiplied by time. The uncertainty of the population exposure value is calculated by taking the 5–95th percentile confidence intervals of 14 GCMs (to reduce the influence of predicted temperature outliers in several GCMs).

## 3. Results

### 3.1. Global Heat-Stress Extreme Distribution

**Figure 2:**
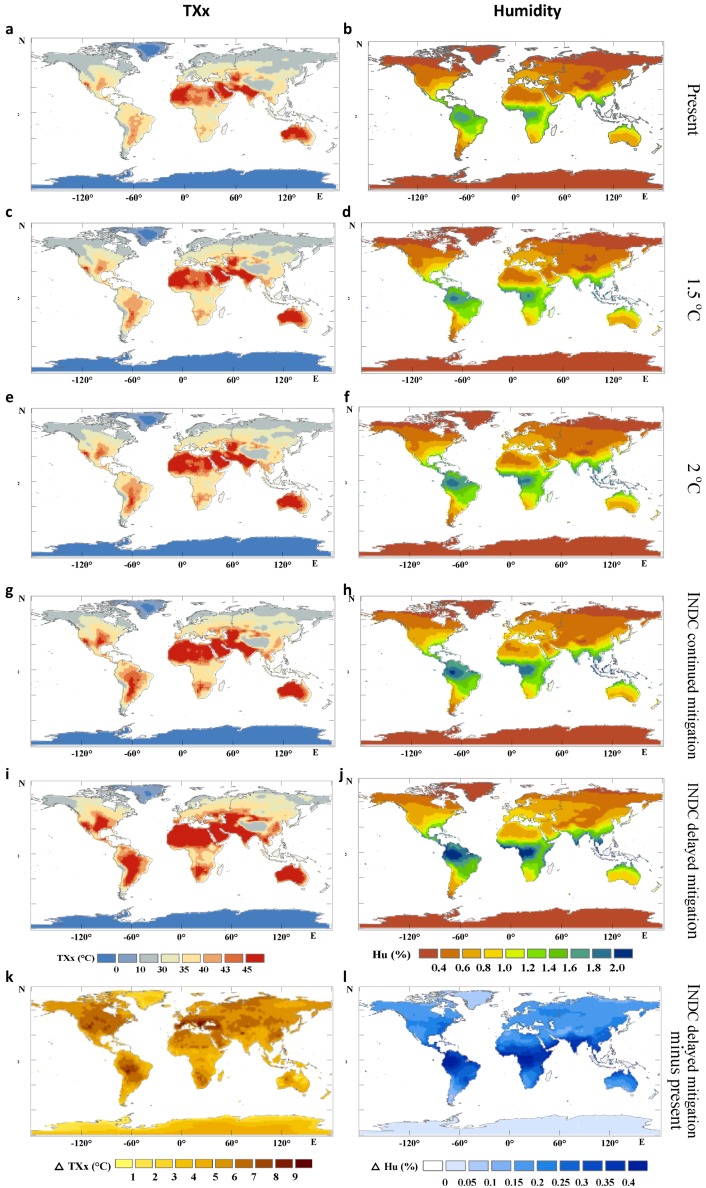
Variation of the maximum atmospheric temperature (TXx, left column) and humidity (specific humidity, right column). (**a,b**): current level; (**c,d**): 1.5 °C scenario; (**e,f**): 2 °C scenario; (**g,h**): INDC continued mitigation scenario; (**i,j**): INDC delayed mitigation scenario; (**k,l**): INDC delayed mitigation scenario minus the current level.

**Figure 3:**
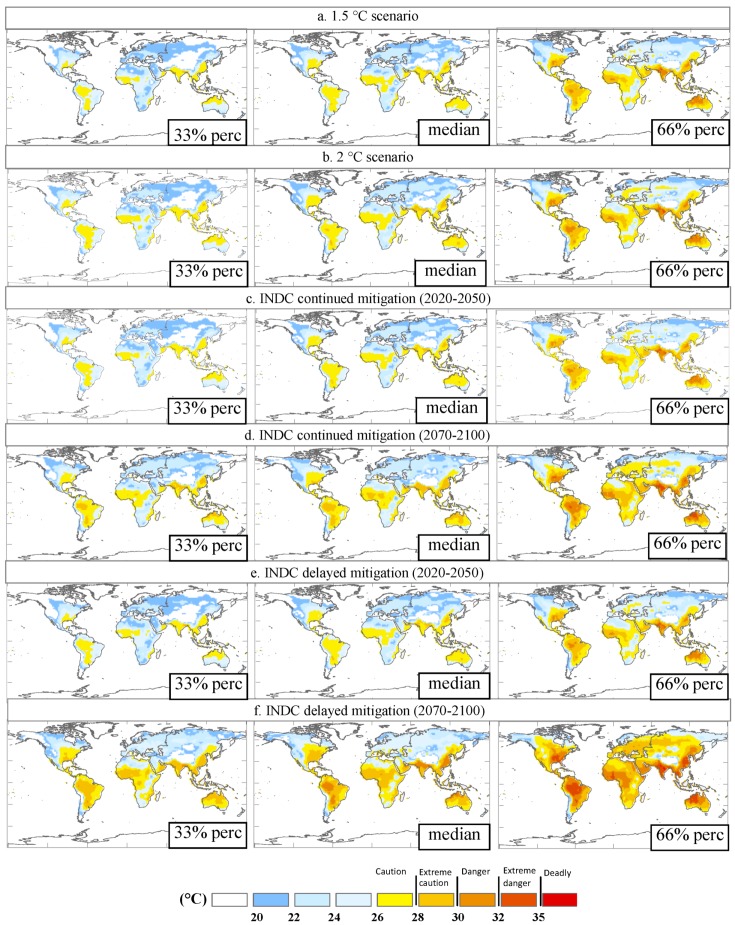
Twmax of the hottest month under (**a**) 1.5 °C scenario, (**b**) 2 °C scenario, (**c**) INDC continued mitigation scenario in 2020–2050, (**d**) INDC continued mitigation scenario in 2070–2100, (**e**) INDC delayed mitigation scenario in 2020–2050 and (**f**) INDC delayed mitigation scenario in 2070–2100. The middle column presents the median of the values simulated by 14 CMIP5 (the fifth phase of the Coupled Model Intercomparison Project) GCMs (generalized circulation models) under four scenarios. The left column presents the 33% quantile of the values simulated by all models. The right column presents the 66% quantile of the values simulated by all models.

Both air temperature and humidity together contribute to the change in heat stress. Figure 2a–l shows the variations of the maximum atmospheric temperature (annual maxima of the daily maximum temperature, TXx, Figure 2 left column) and humidity (annual mean of the daily humidity, Figure 2 right column). The region with the largest temperature increase also often exhibited the greatest change in humidity, thus affecting the prediction of the wet-bulb temperature. Compared to the TXx change, the change in the wet-bulb temperature was slightly less (Figure 3). We used the GCM models ensemble to predict that till to 2020–2050 and 2070–2100 (30-year average) (compared with the current average level during the period 1980–2010), the average $T_{wmax}$ across the whole mid- to low-latitude regions increases approximately 0.5–1.7 °C (10–90% CIs) (2020–2050) and 1.5–3.0 °C (2070–2100) under the INDC continuous mitigation scenario (Figure 3c,d); if policy implementation was weakened, the $T_{wmax}$ for 2070–2100 might increase 3.0–4.5 °C under the INDC delayed mitigation scenario, although the increase for 2020–2050 seems to be close to that with INDC mitigation efforts (Figure 3e,f). However, if limiting warming to a lesser amount (1.5 °C and 2 °C targets), the mean *T_wmax_* would only increase a little bit by approximately 0.5–1.2 °C and 0.8–2.0 °C, respectively (Figure 3a,b).

These projected increases in wet-bulb temperature are predominantly concentrated in some regions. In the delayed mitigation scenario, some regions are particularly vulnerable to heat stress, such as eastern China, South Asia, West Asia, the Amazon, South America, West Africa, the eastern United States, and northern Australia. Among them, South Asia (~34 °C, the highest of all GCMs), East China (the highest of ~33 °C), and South America (the highest of ~33 °C) are the most prominent regions, all of which will exceed their respective historical maximum wet-bulb temperatures. In the INDC continuous mitigation scenario, the heat stress intensity in these hotspot areas is significantly reduced and the average values for these areas are about 1–2 °C. Under the global targets of the 1.5 °C and 2 °C scenarios, the effects of extremely high heat stress can be largely avoided in regions such as West Asia, Australia, and the eastern United States and most models predict that the wet-bulb temperature remains below 29 °C in approximately all regions of the world.

Those regions severely affected by heat stress have different climate and weather patterns during different heat wave events in different scenarios, which could affect the relative importance between temperature and humidity (on the extreme wet-bulb temperature). In some regions, both high temperature and humidity contribute to high wet-bulb temperatures, such as eastern China, South Asia, and the Amazon. Under the delayed and continuous mitigation scenarios, the 30-year average humidity (2070–2100) in these regions increases of 0.25–0.44% and 0.14–0.24%, respectively, compared with the current level and are, respectively, 0.16–0.3% and 0.06–0.11% greater compared with the 2 °C global target scenario. In some regions, the contribution of temperature far exceeds that of humidity, such as West Asia, Australia, and the Sahara. The maximum temperature of these regions may reach 51–53 °C (higher than those in East China, South Asia, and the Amazon) under the continuous mitigation scenario, but the annual humidity is far below that in East China, South Asia, and the Amazon. Therefore, although West Asia, Australia, and the Sahara could experience significant heat-stress effects, they are not as intense as that in East China, South Asia, and the Amazon.

### 3.2. Global Heat-Stress Exposure Frequency 

The duration of heat exposure is critical to determining its effects on health, as any potential respiratory disease response is associated with prolonged heat exposure. To assess the exposure time of the wet-bulb temperature at the regional scale, we predicted the number of days whose wet-bulb temperature may exceed the threshold (the dangerous days, which may have a severe impact on human labor productivity) every year. We selected three regions that are most severely affected by heat stress for analysis.

**Figure 4:**
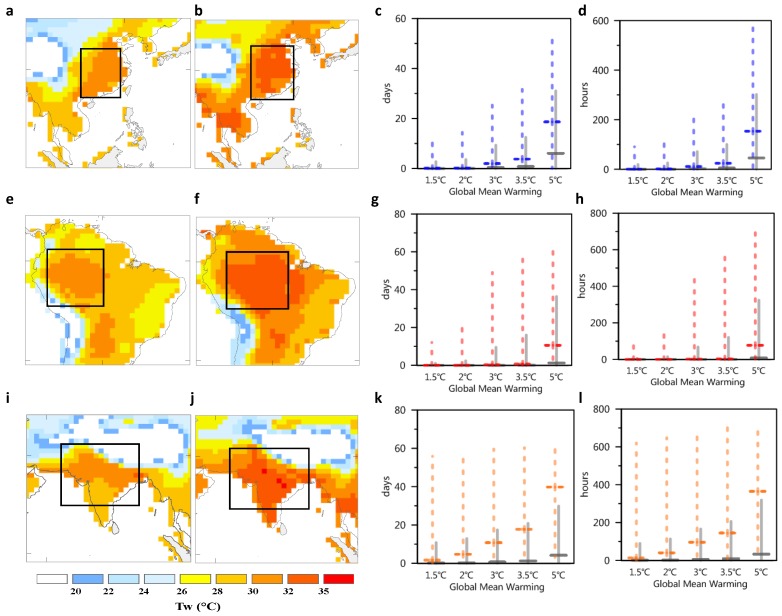
Hotspot regions with high wet-bulb temperatures (two columns on the left) and the duration when the wet-bulb temperature is above 30 °C (two columns on the right). China under the delayed mitigation scenario for 2070–2100: (**a**) Twmax, 50% quantile; (**b**) Twmax, 66% quantile; (**c**) number of days in China; (**d**) number of hours in China. Amazon under the delayed mitigation scenario for 2070–2100: (**e**) Twmax, 50% quantile; (**f**) Twmax, 66% quantile; (**g**) number of days in the Amazon; h, number of hours in the Amazon. South Asia under the delayed mitigation scenario for 2070–2100: (**i**) Twmax, 50% quantile; (**j**) Twmax, 66% quantile; (**k**) number of days in South Asia; (**l**) number of days in South Asia. In the two columns on the right, colored dotted lines represent the maximum values of grid points predicted by multiple models, and the solid gray line represents the average of all grid points predicted by multiple models.

In East China (Figure 4a,b), for the period 2070–2100, the number of days whose wet-bulb temperature may exceed 30 °C may be 5 to 20 days per year for most areas (under the delayed mitigation scenario) (Figure 4c), whereas it might be 15 to 50 days in North China area, which is related to the high humidity caused by irrigation in North China [32]. Under the INDC continuous mitigation scenario, the number of days with heat stress is reduced to 1 to 5 days per year, and only the prediction of some sensitive models (such as CanESM2) reaches 25 to 30 days. In terms of the total hours of exposure (Figure 4d), the number of hours whose wet-bulb temperature may exceed 30 °C might be 45–150 hours per year (under the delayed mitigation scenario) and 5–25 hours (under the INDC continuous mitigation scenario). Compared with the delayed mitigation scenario, continuous mitigation efforts can significantly shorten the number of days and hours of heat exposure in North China; however, compared with the 1.5 °C and 2 °C global target scenarios, continuous mitigation efforts may still lead to certain heat exposure risks. Under the 1.5 °C and 2 °C global target scenarios, the heat exposure risk in North China is low.

In the Amazon region (especially in the northwestern region of Brazil) (Figure 4e,f), for the 30-year average of the period 2070–2100, the number of days whose wet-bulb temperature may exceed 30 °C might be 1 to 10 days per year (under the delayed mitigation scenario) (Figure 4g). Under the INDC continuous mitigation scenario, the number of days with heat stress each year is greatly reduced. The median values simulated by modeling continuous mitigation efforts is comparable to that under the 1.5 °C and 2 °C global target scenarios; in other words, the INDC continuous mitigation efforts may play an important role in the reduction of heat stress in the Amazon region.

In South Asia, especially in India and Pakistan (Figure 4i,j), heat-stress exposure time is the longest (compared to the previous two regions). For the period 2070–2100, the number of days whose wet-bulb temperature may exceed 30 °C and might be 4 to 30 days per year (under the delayed mitigation scenario) (Figure 4k), whereas that along the India-Pakistan border and India’s eastern coast may reach 40 days (several models predict 60 days). This is related to the high humidity caused by the large local agricultural land area and the high irrigation level. Under the INDC continuous mitigation scenario, the number of days with heat stress each year is reduced to 1 to 18 days, and predictions of some highly sensitive models may also reach 60 days. In terms of the total exposure time, the wet-bulb temperature may exceed 30 °C at 400 hours per year, under the delayed mitigation scenario, or over 100 hours, under the INDC continuous mitigation scenario (Figure 4l). Although continuous mitigation efforts (compared to the delayed mitigation scenario) can significantly shorten the days and hours of heat exposure in India, it may still lead to some heat-exposure risks. Only under the 1.5 °C global target scenario can the heat exposure risk in India be reduced to its lowest level.

**Figure 5:**
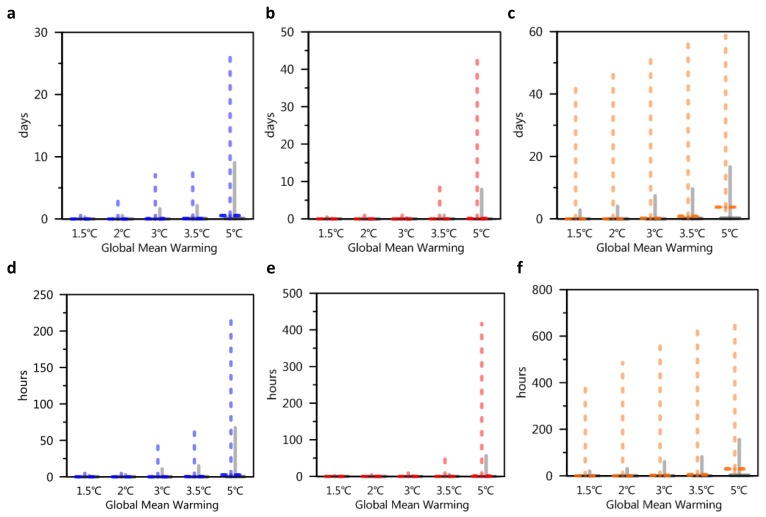
Number of days and hours when the wet-bulb temperature exceeds 32 °C. (**a**) Days in China; (**b**) days in the Amazon; (**c**) days in South Asia; (**d**) hours in China; (**e**) hours in Amazon; (**f**) hours in South Asia. The colored dashed lines in each graph represent the maximum values of grid points predicted by multiple models, and the solid gray lines represent the average of all grids predicted by multiple models.

In addition, we also predicted the number of days whose wet-bulb temperature may exceed the threshold (which may have an extremely severe impact on human life) every year. Many studies have suggested that temperatures exceeding 32 °C could have a harmful effect on resting people (resting without labor) [33,34]. For the period 2070–2100, in India, the number of days whose wet-bulb temperature might exceed 32 °C might be several days to several tens of days per year (under the delayed mitigation scenario); however, under the INDC continuous mitigation scenario, the number of days with a temperature over 32 °C is significantly reduced by one order of magnitude. Compared to India, China and the Amazon have less number of days with temperatures over 32 °C per year under the delayed mitigation scenario (less than one order of magnitude) (Figure 5a–f).

### 3.3. Global Population Exposure to Critical Heat Events

**Figure 6:**
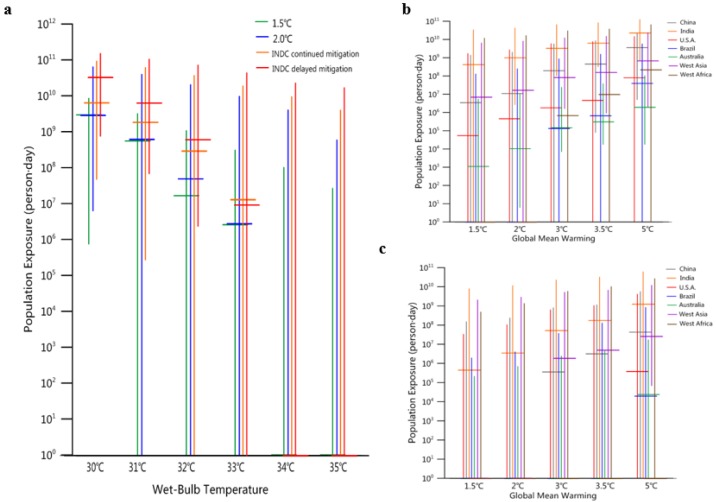
Annual global person-days exposed to different wet-bulb temperature thresholds. (**a**) Annual global exposure under the four scenarios at wet-bulb temperatures of 30–35 °C. The error bars (vertical lines) present the range of 5–95% for 14 GCMs. The horizontal lines indicate the median values for 14 GCMs. Right: Annual exposure of regions with wet-bulb temperatures over (**b**) 30 °C and (**c**) 32 °C (32 °C is approximately the upper threshold of sustainable labor [33,34]). For the exposures at 34 °C and 35 °C of extreme high thresholds in (**a**), only a few models simulate the values, so the median values from 14 models are very small. Some models (such as IPSL-CM5A-LR) simulate the extremely lower value than other models results, which causes the low end of some error bars to be close to zero.

Large population growth is expected throughout the 21st century, especially in developing countries. Most of this increase is expected to occur in areas with high wet-bulb temperatures, leading to a significant increase in exposure to dangerous high-temperature conditions. For the period 2070–2100 (30-year average), we used SSP population prediction data to estimate the number of person-days (the number of days and number of people superimposed) exposed to high wet-bulb temperatures (Figure 6). By using 14 GCMs under the four emission scenarios, we estimated the extensive exposure uncertainty. The uncertainty is assumed to be caused by GCM variability and the uncertainty of future emission trajectories.

The population exposed to extreme wet-bulb temperatures is largely determined by future greenhouse gas emissions. Figure 6a shows the annual exposure predicted by the 14 GCMs (wet-bulb temperature range of 30–35 °C) under the delayed and continuous mitigation scenarios and the 1.5 °C and 2 °C global target scenarios. In the wet-bulb temperature range of 30–33 °C, the population exposure differs significantly between the INDC scenarios and the 1.5 °C and 2 °C global target scenarios by several orders of magnitude (person-days). The population exposed to wet-bulb temperatures over 30 °C and 32 °C under the INDC continuous mitigation scenario (compared to the delayed mitigation scenario) decreases by approximately one order of magnitude; however, compared with the 1.5 °C and 2 °C global target scenarios, exposure increases by half and one order of magnitude (wet-bulb temperatures of 30 °C and 32 °C, respectively). Wet-bulb temperatures of 34 °C and 35 °C are very rare, and only individual models have simulation results.

Figure 6b,c, respectively, present the population exposure when the wet-bulb temperature is over 30 °C and 32 °C at the regional scale. For the period 2070–2100, South Asia (India) is the area with the greatest population size exposed to the 30 °C wet-bulb temperature (approximately 3 × 10^9^ to 2.3 × 10^10^ person-days for the INDC scenarios), followed by East China, West Asia, Saudi Arabia, West Africa (Ghana, Côte d’Ivoire, etc.), the eastern United States, Brazil, and northern Australia. In the 1.5 °C and 2 °C global target scenarios, the population exposure in each country is significantly reduced by one to three orders of magnitude. For example, India’s exposure is approximately 4 × 10^8^ to 1 × 10^9^ person-days under the 1.5 °C and 2 °C global targets scenarios; West Asia’s exposure is approximately 6.7 × 10^6^ to 1.6 × 10^7^ person-days; and East China’s exposure is approximately 3 × 10^6^ to 1 × 10^7^ person-days.

Compared with populations of different regions exposed to wet-bulb temperatures of 30 °C, the populations of different regions exposed to 32 °C is significantly less (there is approximately one or two orders of magnitude decrease in each region). The region with the largest population exposure is India (for the INDC scenarios, exposure is approximately 5.2 × 10^7^ to 1.2 × 10^9^ person-days), followed by East China, West Asia and the eastern United States

## 4. Discussion

Previously, when studying the human health risks of heat waves, only a few studies focused on the humidity issues associated with climate change. However, humidity may prove a breaking point for some areas as the temperature rises. As global temperatures increase, the atmosphere can accommodate more water vapor, which can greatly amplify the influence of heat. Generally, humid heat is more oppressive than “dry” heat for humans. This is because humans cool their bodies through sweating and evaporation from the skin into the air; when the air is already full of water, evaporation from skin slows, eventually becoming impossible. Therefore, the combined changes in both the temperature and humidity pose a major threat to human health. Similar threats would also affect other animals and vegetations. For instance, heat stress may affect directly and indirectly dairy cows’ metabolic and physiological acclimation. This may reduce the synthesis of milk and makes dairy cows more susceptible to illness [35,36]. Currently, there is an increasing concern of the heat stress on animal production and welfare in both temperate and tropical areas [37]. In addition, heat stress affects plant growth throughout its ontogeny, which may slow down or inhibit seed germination depending on plant species and the intensity of the stress [38].

This study used the global climate models ensemble to predict the wet-bulb temperature (depending on the combination of heat and humidity) under various mitigation efforts presented in the Paris Agreement, to reflect the impact of different mitigation efforts. The study found that for the period 2070–2100, under the delayed mitigation scenario, many areas of the world (e.g., East China, India-Pakistan border area, West Asia, the Amazon, West Africa, the eastern United States, northern Australia) may be exposed to high heat stress. The heat stress temperature in the most severe areas might reach 34 °C. If countries can maintain their mitigation commitments and steadily implement mitigation efforts, then the heat stress intensity in these hotspot areas will be significantly reduced. If mitigation efforts can further achieve the 2 °C global temperature control goal, then extreme heat stress in West Asia, Australia, the eastern United States, and other regions can possibly be avoided, and the duration of extreme heat stress can be shortened in those regions where extreme heat stress is inevitable (e.g., India). We believe that hotspot regions affected by heat stresses could greatly benefit from enhanced global mitigation efforts because these regions have few cooling facilities, weak adaptability and are areas with the highest population density.

Indoor experiments have shown that the wet-bulb temperature of 32 °C is an upper threshold for many people participating in normal outdoor activities. Many people collapse before reaching the wet-bulb temperature of 32 °C or any temperature close to that level. This level is rarely reached anywhere today. However, our study shows that by the end of the 21st century, if mitigation efforts slow, some regions (the Amazon, West Asia, eastern China) may experience up to ten days or more per year at this level. South Asia’s exposure could amount to more than 30 days per year, and hundreds of millions of people worldwide would suffer as a result. If mitigation actions steadily progress, some underdeveloped, densely populated areas might avoid the worst impact. Although such weather conditions might not directly kill people or stop all activities, farm labor or other outdoor activities under such circumstances could lead to chronic kidney disease and other harmful health effects. Certainly, low-latitude regions would suffer the greatest losses.

## 5. Conclusions

This study used the GCMs ensemble to analyze the regional heat-stress changes for the national pledged emission reduction scenarios under the Paris Agreement and also compared the heat stress results with the 1.5 °C and 2 °C global target scenarios. The response of regional heat stress to different emission scenarios has large differences. The primary research results are summarized as follows:

1. With the combination of temperature and humidity under climate change, heat stress intensity in the middle- and low-latitude regions will increase significantly. Under the delayed mitigation scenario, by the end of the 21st century, eastern China, South Asia, the Amazon, western Africa, the eastern United States, northern Australia, and other regions will be particularly vulnerable to heat stress; among them, South Asia, eastern China, and the Amazon are the most vulnerable. While under the INDC continuous mitigation efforts, the wet-bulb temperature of these three regions would decrease 1–2 °C in 2070–2100. Under the 1.5 °C and 2 °C global target scenarios, the predicted wet-bulb temperature of most regions, according to the majority of the models is below 29 °C.
2. The frequency of exposure to the dangerous wet-bulb temperature threshold (which may severely affect human labor productivity) also potentially increases. In particular, the heat-stress exposure duration is the longest along the India-Pakistan border region of South Asia, and temperatures exceeding 30 °C and 32 °C during the period 2070–2100 are expected to occur more than 40 days and several to ten days per year, respectively (under the delayed mitigation scenario). Continuous mitigation efforts (compared to the delayed mitigation scenario) can significantly shorten the days and hours of heat exposure in India, but compared with the 1.5 °C and 2 °C global target scenarios, continuous mitigation efforts may still lead to higher exposure risks of heat stress.
3. Populations exposed to dangerous wet-bulb temperatures are expected to continue to increase with climate change. More active mitigation policies could reduce population exposure (to wet-bulb temperatures greater than 30–32 °C) by approximately one order of magnitude (pursuing the INDC continuous mitigation scenario versus the delayed mitigation scenario), and half to one order of magnitude (the 2 °C global target scenario versus the INDC continuous mitigation scenario). Given the dramatic increase in the number of people worldwide who may be exposed to dangerous heat stress, failure to take proactive mitigation and adaptation measures in the future will likely result in greater economic losses and increased heat-related mortality.

## Supplementary Materials

The following are available online at https://www.mdpi.com/1660-4601/16/12/2202/s1, Table S1: Detailed information about the INDCs dataset, Table S2: Earth System Models used in this study.
