# Supplementary material for: Heat Stress Response to National-Committed Emission Reductions under the Paris Agreement
Source: Int J Environ Res Public Health. 2019 Jun 21;16(12):2202. doi: 10.3390/ijerph16122202 (PMC6616907; doi:10.3390/ijerph16122202)
Supplement: Supplementary file 1 [file ijerph-16-02202-s001.pdf]

S1 Detailed information about the INDCs dataset

The INDCs provide emission targets before 2030. Table S1, from the study of Wang [2018], shows the national mitigation objectives submitted to INDCs to date. Simulations on future emissions from 28 socioeconomic models are used to extend the INDC scenario to the end of this century. We considered carbon removal rate, carbon capture and storage (CCS) technology, energy structure improvement, and carbon neutralization time. Here, we consider many possibilities of extending the “Mitigation Action of INDCs”. According to the IPCC AR5 scenario database, we used scenarios that met the 2030 greenhouse gas emission level consistent with the INDC (50–56 Gt CO_2 eq/yr) scenario, assuming that there is no sudden change in climate action during the 21st century. Considering the difficulty and uncertainty of future carbon removal technologies, we adopted a conservative method for the future availability of negative emission technologies and eliminate the scenario where CCS > 15 Gt CO_2 eq/yr.

Based on some key characteristics (e.g., emission targets in specific years, renewable energy structure, and number of CCS technologies), these emission pathways are further divided into six groups, as shown in Figure 1. Group I (baseline scenario) contains scenarios without any additional climate policies or mitigation actions, where the greenhouse gas emissions continue to increase according to current trends. Group II is similar to the baseline scenario but allows for lower energy intensity in the future. Group III represents a weak-policy baseline scenario considering existing climate policies, a weak interpretation (e.g., 2020 Copenhagen Pledges), and extrapolation of these targets beyond 2020 based on emissions intensity. Global emissions were assumed to peak in 2030 in Groups IV to VI. Specifically, Group IV may be described as a “continued action” pathway. The relatively constant decarbonization rates were approximately followed for the period after 2030. The overall trend of Group V is similar to Group IV, but more rapid mitigation after 2030 is the distinguishing characteristic. Group VI involves CCS action accelerating decarbonization and determining negative emissions in some pathways.

In our discussion of the heat stress response in the global INDC scenario, we referred to the first group as the "delayed action" scenario and the third and fourth groups as the extended "continuous action" (INDC commitment) scenario. The heat stress responses at the 1.5 °C and 2 °C warming levels were compared with the heat stress response s under the INDC scenarios.

S2 Earth System Models used in this study

**Model**	**Institute**	**Country**	**Horizontal Resolution**
BCC-CSM1.1	Beijing Climate Center	China	128×64
BCC-CSM1.1(m)			320×160
CanESM2	Canadian Centre for Climate Modelling and Analysis	Canada	128×64
CESM1(BGC)	National Center for Atmospheric Research	USA	288×192
HadGEM2-CC	Met Office Hadley Centre	UK	192×145
HadGEM2-ES			192×145
IPSL-CM5A-LR	Institute Pierre Simon Laplace	France	96×96
IPSL-CM5A-MR			144×143
IPSL-CM5B-LR			96×96
MIROC-ESM	Atmosphere and Ocean Research Institute (University of Tokyo), National Institute for Environmental Studies, and Japan Agency for Marine-Earth Science and Technology	Japan	128×64
MIROC-ESM-CHEM			128×64
MPI-ESM-LR	Max Planck Institute for Meteorology	Germany	192×96
MPI-ESM-MR			192×96
NorESM1-ME	Norwegian Earth System Model	Norway	144×96

Table S2. Basic information of 14 CMIP5 models applied in this research.
